# Incidence and predictors of acute kidney injury among traumatic brain injury patients in Northwest Ethiopia: a cohort study using survival analysis

**DOI:** 10.1186/s12882-025-04024-3

**Published:** 2025-02-25

**Authors:** Zelalem Alamrew Anteneh, Semew Kassa Kebede, Abebaw Gedef Azene

**Affiliations:** 1 Department of Epidemiology and Biostatistics, School of Public Health, College of Medicine and Health Science, Bahir Dar University, Bahir Dar, Ethiopia; 2 Division of Epidemiology and Biostatistics, School of Public Health, Faculty of Health Sciences University of Witwatersrand, Johannesburg, Republic of South Africa

**Keywords:** Acute kidney injury (AKI), Traumatic brain injury, Predictors of AKI, Ethiopia

## Abstract

**Background:**

Acute kidney injury (AKI) is a sudden and reversible decrease in kidney function, causing the retention of waste products in the blood and potentially resulting in severe complications or death if not timely managed. Studies on AKI among traumatic brain injury patients in low-income nations like Ethiopia is very critical due to the limited healthcare resources, high burden of trauma-related injuries, and lack of robust data on the incidence and risk factors of AKI in such settings, which hinders effective prevention and treatment strategies tailored to these vulnerable populations Therefore, this study aimed to assess the incidence and predictors of AKI among traumatic brain injury patients.

**Methods:**

A retrospective cohort study was conducted among 450 adult patients with traumatic brain injuries admitted to Tibebe-Ghion Specialized Hospital in Ethiopia. Kaplan- Meir curve and Log rank test were used to estimate and compare survival probability of different categories. A multivariable Cox proportional hazards model was used to identify determinants of acute kidney injury (AKI).

**Results:**

The incidence of AKI was 10.9%, with a median follow-up period of 42 days. Significant predictors of AKI among traumatic brain injury patients included age (AHR: 1.05, 95% CI: 1.02–1.07), severe head injury (AHR: 1.46, 95% CI: 1.02–2.09), unreactive pupillary response (AHR: 4.82, 95% CI: 1.82–12.72), and hypotension (AHR: 3.45, 95% CI: 1.71–6.96).

**Conclusions:**

The study found that AKI occurs in more than one in ten patients with traumatic brain injuries, with significant predictors including older age, severe head injury, unreactive pupillary response, and hypotension. These findings highlight the need for careful monitoring and early intervention for high-risk patients to prevent AKI and improve overall outcomes. Implementing targeted prevention and treatment strategies in settings with limited resources can help mitigate the burden of AKI and enhance patient care in vulnerable populations.

## Background

Traumatic brain injury (TBI) is a major public health issue and a leading cause of mortality and morbidity globally, particularly in low- and middle-income countries. Annually, approximately 10 million people worldwide suffer from TBI, with acute kidney injury (AKI) occurring in up to 50% of trauma patients, often resulting in prolonged hospital stays and increased mortality [1–3]. Globally, 90% of trauma-related deaths occur in low- and middle-income countries (LMICs), with half of these fatalities resulting from central nervous system injuries. In LMICs, mortality rates for patients with traumatic brain injury (TBI) are more than twice as high as those for patients in high-income countries [4, 5].

Acute kidney injury (AKI) is a common complication in patients with severe trauma, particularly those with TBI, and is linked to increased morbidity and mortality [6, 7]. Worldwide, approximately 13.3 million people are affected by AKI annually, resulting in an estimated 1.7 million deaths each year [8, 9]. AKI is characterized by a rapid decline in kidney function, encompassing both structural damage and functional impairment that leads to the accumulation of nitrogenous waste products. It is diagnosed based on elevated serum creatinine levels and decreased urinary output (oliguria) [10–12].Patients with major trauma are at risk for AKI due to various factors, including systemic inflammation, hypovolemic shock, massive transfusion, rhabdomyolysis, abdominal compartment syndrome, and major surgery [6, 13, 14]. The incidence of AKI among TBI patients is notably high, especially among older patients and those with lower Glasgow Coma Scale (GCS) scores, with an AKI occurrence rate of up to 12% [7, 15].

Separating TBI patients from other trauma patients in the context of acute kidney injury (AKI) is crucial due to unique TBI-related factors. TBI can cause physiological disruptions like paroxysmal sympathetic hyperactivity (PSH), increasing metabolic demand and renal stress [16]. TBI patients also have higher susceptibility to global ischemia and hypoxemia, affecting renal perfusion [17]. The use of nephrotoxic medications is more common in TBI patients due to the management of associated complications, and infectious complications are more frequent, further compromising kidney function. These combined factors elevate the risk of AKI specifically in TBI patients compared to other trauma patients. Several predictors contribute to the occurrences of AKI among adults with traumatic brain injury (TBI). These include socio-demographic factors, such as age and gender, patient-related characteristics like comorbidities, and clinical variables including the severity of TBI and the timing of interventions [18–21].

In Ethiopia, the number of road traffic accidents and related deaths is high, driven by the country’s rapidly growing economy and the increasing rates of road construction and civil war are some of leading causes of TBI; in turn this is the main causes of AKI. However, data on the incidence and predictors of AKI following TBI is scarce; therefore, quantifying the incidence and identifying predictors of AKI is crucial for preventing AKI in TBI patients, which can help reduce the growing burden of end-stage kidney disease, decrease the need for dialysis, and lower mortality rates. Therefore, this study aimed to assess the incidence and predictors of AKI among TBI patients.

## Methods and materials

### Study area and period

The study was conducted at Tibebe Ghion Specialized Hospital, located in Bahir Dar, the capital city of the Amhara region, approximately 550 km from Addis Ababa, Ethiopia’s capital. The hospital serves a population of over 10 million people from both Bahir Dar and surrounding areas. As a teaching hospital affiliated with Bahir Dar University, it has more than 500 inpatient beds, including 7 main wards, 2 ICU rooms, and over 126 clinical service rooms. Tibebe Ghion Specialized Hospital does not offer renal replacement therapy or dialysis, so patients requiring these services are referred to other facilities for appropriate care. This study utilized four years of health record data from adult traumatic brain injury patients aged 15 and older, admitted to the hospital between October 2018 and August 2022.

### Study design

This study used a hospital-based retrospective cohort study design aiming for analyzing the time-to-event data to predict the incidence of AKI and to find out predictors of AKI among Traumatic Brain Injury (TBI) patients in Tibebe Ghion Specialized hospital surgical ward and ICU using survival analysis techniques.

### Study population

The study population includes all adult TBI patients admitted to the hospital between October 2018 and August 2022. Patients were followed from the time of TBI diagnosis upon hospital admission until the occurrence of AKI or the end of the study period (censoring). Each TBI patient was followed until the development of AKI (event) or end of the hospital stay without developing AKI (censored) from the date of their hospital admission.

### Eligibility criteria

The study included all TBI patients aged 15 years and older admitted to the ICU and surgical wards at Tibebe Ghion Hospital from October 2018 to August 2022.

### Outcome variables

Primary outcomes:

The occurrence (incidence) of Acute kidney injury (Yes/No, or 1/0)- indicating whether or not a TBI patient developed AKI during the study period. And

Time to AKI occurrence: The number of days to the occurrence of AKI from the time of TBI diagnosis following hospital admission.

Censoring: TBI patients who didn’t develop AKI during the hospital stay (follow-up period) were censored observations.

### Independent variables

**Socio demographic factors**: Age, Sex, Residence.

**Clinical factors**: Complication after admission, Glasgow coma scale, Severity of head injury, Types of head injury, Hypernatremia, pupillary reactivity, Hospital length of stay.

**Intervention factors**: Osmotic therapy, RBC transfusion, ICU admission.

**Patient related factors**: Co-morbidity, Mechanism of injury.

As this study involved survival analysis, hypothesis of the study:

#### Null hypothesis (H0)

The survival of patients with AKI among TBI patients does not differ significantly with respect to identified predictors

#### Alternative hypothesis (H1)

The survival status of patients with AKI among TBI patients differs significantly with respect to identified predictors

### Operational definition

This study used the hospital physicians’ working definition for AKI based on Improving Global Outcomes (KDIGO) clinical practice guidelines. Acute Kidney Injury (AKI) is said to occur if any one of the following criteria happening:

Increase in Serum Creatinine (SCr): Absolute increase in SCr by ≥ 0.3 mg/dL (26.5 µmol/L) within 48 h. OR, Relative increase in SCr to ≥ 1.5 times the baseline value within the prior 7 days.

Reduction in Urine Output: Urine volume < 0.5 mL/kg/hour for at least 6 h [12].

Traumatic brain injury (TBI): categories mild, moderate, and severe based on the Glasgow Coma Scale (GCS), the duration of loss of consciousness (LOC), and the duration of post-traumatic amnesia (PTA). This is classification is for the sake of intervention and clinical management.

**GCS**: The Glasgow coma scale is a scale used for assessing the neurological status of the patient and to classify head injury as; mild TBI GCS (13–15), moderate TBI (9–12), or severe TBI < 9.

**Elevated intracranial pressure**: in this study, elevated intracranial pressure (ICP) was defined as a sustained ICP value greater than 20 mmHg, otherwise, normal.

**Event**: was the occurrence of AKI among TBI from admission to the end of study.

**Censored**: was defined as those TBI patients who did not develop the outcomes of interest at the end of study period.

**Time to event**: a time between admission and occurrence of AKI.

**Median survival time**: a time at which half of patients suffered AKI.

**Stick injury**: A stick injury is an injury commonly occurring during acts of violence or assault, intentionally inflicted during confrontations where a stick or similar object is used as a weapon.

### Sample size determination and sampling procedure

We determined the sample size for our study using STATA version 14 software, taking into account a previous study, hazard ratios, and survival probabilities for both exposed and non-exposed groups, with a significance level of α = 5% and a power of 80%. Accordingly, we planned to include a total of 450 participants in the analysis to address our study objectives.

### Sampling technique and procedure

The patient charts were selected by using simple random sampling technique from registration book of adult ICU, and surgical ward in TGSH. This was done using registration book of the past five years in adult ICU, and surgical ward and the selected medical registration number of these patients card was collected from the card room.

### Data collection tool and procedure

The data was collected by record review using structured data extraction checklist from September to November 2022. The data extraction checklist had socio-demographic, clinical patient related and institution related variables and data’s were collected for all subjects in the sample from patient cards. The data was collected from selected cards by semi-structured data extraction checklist. Before data collection two BSc Nurses for data collection and one MSc. Nurse for supervision were recruited. One day training was provided for data collectors and a supervisor about the meaning of every item of questionnaire and the techniques of data collection.

### Data quality control

Before the actual data collection pretest was conducted on 5% of the sample size in TGSH, two weeks before the actual data collection. The pretest was analyzed and interpreted final modification was done on data extraction tool. Quality of data was assured by training data collectors and supervisors, monitoring the data collection process and checking completeness of data during collection time. The data was entered into epi data version 3.1 and cleaned by principal investigator before analysis.

### Data processing and analysis

Data was coded and entered into Epi data version 3.1 exported to stata version 14 for analysis. A descriptive statistical summary was used to describe socio-demographic, institution, clinical, and patient-related variables of the study. The occurrence of AKI Incidence was calculated by dividing the total number of incident cases of AKI to the total person year of follow-up for the study period.

The log rank test and Kaplan- Meir survival curve was used to compare different survival probability and Cox-proportional hazard regression model was used to see the association of each independent variable with the AKI. PH assumption was checked graphically and with shoenfeld residual test and goodness of fit of the model was assessed using Cox Snell residual. A pair wise correlation test and variance inflation factor (VIF) was done to check presence of Multicollinearity between each independent variable.

Bivariable Cox regression analysis was conducted to assess the relationship within each predictor variable and TBI related AKI. Variables that have P-value of ≤ 0.2 with TBI related AKI in Bivariable Cox proportional regression model were entered to the multivariable Cox-proportional hazards regression analysis model. In multivariable model, a variable with P-value of less than 0.05 was taken as to declare statistically significant association between predictors and AKI among TBI.

## Results

### Socio-demographic characteristics

**Fig. 1:**
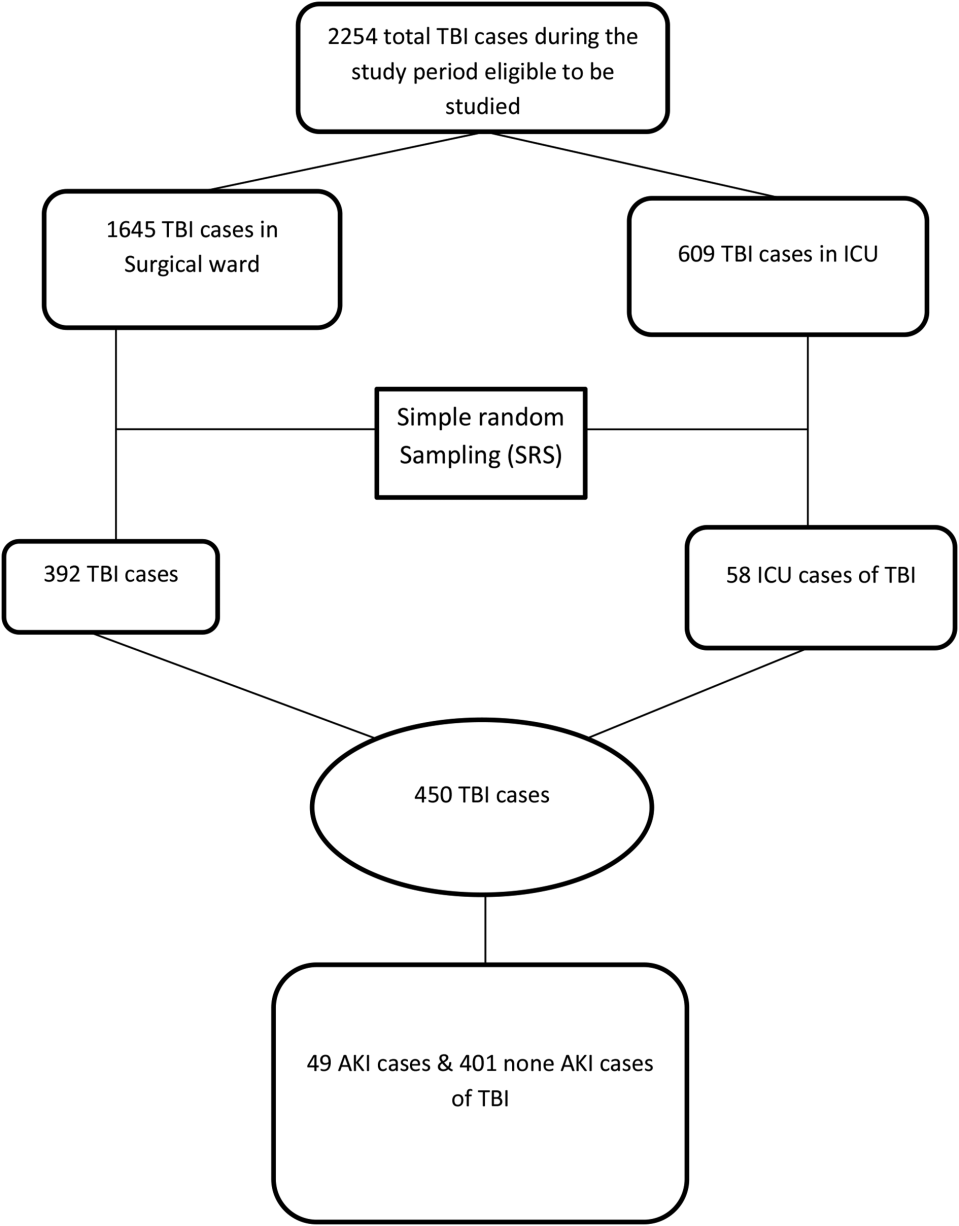
Flow diagram of Traumatic Brain Injury (TBI) patients at TGSH in Bahir Dar city, Northwest Ethiopia, 2022

In this study, there were 2254 TBI registered cases (609 ICU admitted, and 1645 surgical ward admitted) TBI patients considered for sampling in the study period. A total of 450 TBI patients (58 ICU and 392 surgical wards) TBI patient charts were selected, reviewed and included in our analysis. See flow chart (Fig. 1). The median age of patients at the time of admission was 40 years, with an interquartile range (IQR) of 28 to 52 years. Among the patients with TBI, 349 (77.56%) were males. And 295 (65.56%) of the TBI patients included in the study were from rural areas. Regarding the mechanism of injury, majority of patients who developed AKI after TBI were with Road Traffic Accident (RTA) 22(14.10%) followed by stick injury 12(7.84%). Among the 450 TBI patients, 53 (11.78%) had diabetes mellitus (DM), and 12 (2.67%) had hypertension (HTN) as comorbid conditions (Table 1).

**Table 1:** Baseline socio-demographic and patient related characteristics of the study participants

Variables	Category	Total *N* (%)	Non-AKI *n* = 401 *N* (%)	AKI *n* = 49 *N* (%)
Sex	Male Female	349(77.56%) 101(22.44%)	314(89.97%) 87(86.14%)	35(10.03%) 14(13.86%)
Address	Rural Urban	295(65.56%) 155(34.44%)	265(89.83%) 136(87.74%)	30(10.17%) 19(12.26%)
Mechanism of injury	Fall down Stick RTA Bullet	115(25.56%) 153(34.00%) 156(34.66%) 26(5.78%)	106(92.17%) 141(92.16%) 134(85.89%) 20(76.92%)	9(7.83%) 12(7.84%) 22(14.10%) 6(23.07%)
DM	Yes No	53(11.78%) 397(88.22%)	27(50.94%) 374(94.00%)	26(49.05%) 23(5.00%)
Hypertension	Yes No	12(2.67%) 438(97.33%)	9(75.00%) 392(90.95%)	3(25.00%) 46(9.05%)

Among the 450 patients, the majority, 213 (47.33%) were classified as having mild head injuries, followed by 158 (35.11%) with moderate head injuries. Most patients, 333 (74.00%) had bilaterally reactive pupils, and more than two-thirds had penetrating head injuries. Regarding complications at the time of admission, 93 (20.66%) of TBI patients experienced elevated intracranial pressure, and 57 (12.68%) developed aspiration pneumonia. Out of 450 patients, 58(12.89%) of the study participants were admitted to ICU and 108(24.00%) had osmotic therapy to decrease intracranial pressure elevation. And 46(10.22%) of the patients had history of blood transfusion during their hospitalization due to TBI (Table 2).

**Table 2:** Clinical and Intervention-Related factors of study participants

Variables	Category	Total *N* (%)	Non-AKI *N* (%)	AKI *N* (%)
Hypernatremia	Yes No	21(4.67%) 429(95.33%)	16(76.19%) 385(89.74%)	5(23.81%) 44(10.25%)
Types of injury	Blunt penetrating	137(30.44%) 313(69.56%)	123(89.78%) 278(88.82%)	14(10.22%) 35(11.18%)
Subdural hematoma	Yes No	181(40.22%) 269(57.78%)	149(82.32%) 252(93.96%)	32(17.67%) 17(6.32%)
Hemorrhagic contusion	Yes No	102(22.67%) 348(77.33%)	74(72.54%) 327(93.96%)	28(27.47%) 21(6.03%)
Co-existed injury	Yes No	149(33.11%) 301(66.89%)	123(82.55%) 278(92.36)	26(17.45%) 23(7.64%)
SBP	< 90 90–140 > 140	32(7.11%) 396(88.00%) 22(4.88)	12(37.50%) 371(93.69%) 18(81.81%)	20(62.50%) 25(6.31%) 4(18.18%)
CT scan	Yes No	402(89.33%) 48(10.67%)	354(88.06%) 47(97.91%)	48(11.94%) 1(2.08%)
Aspiration pneumonia	Yes No	57(12.68%) 393(87.33%)	30(52.63%) 371(94.40%)	27(47.36%) 22(5.59%)
ICP	Yes No	93(20.66%) 357(79.33%)	63(67.74%) 338(94.67%)	30(32.25%) 19(5.32%)
Osmotic therapy	Yes No	108(24.00%) 342(76.00%)	78(72.22%) 323(94.44%)	30(27.78%) 19(5.56%)
ICU admission	Yes No	58(12.89%) 392(87.11%)	28(48.27%) 373(95.15%)	30(51.72%) 19(4.85)
RBC transfusion	Yes No	46(10.22%) 404(89.78%)	37(80.43%) 364(90.09%)	9(19.56%) 40(9.90%)

### Incidence of acute kidney injury

The occurrence of acute kidney injury (AKI) in the cohort, observed over 6,564 person-days was 7.46 (95% CI: 5.64–9.88) per 1,000 person-days. Of the total 450 TBI patients included in the analysis, the overall cumulative incidence of AKI was 49(10.9%) during the study period. The median survival time to develop AKI among patients admitted with traumatic brain injury was 42 days with an interquartile range (IQR) of 31–52 days.

The Kaplan-Meier survival curve estimation indicated that the cumulative survival probability after admission to TGSH was 0.3204 (95% CI: 0.1397–0.5178). The estimated cumulative survival probability was 96.88% (95% CI: 94.27–98.32) within the first 10 days, 94.78% (95% CI: 91.41–96.85) at two weeks, and 75.11% (95% CI: 65.60-82.34) at 30 days of follow-up (Table 3).

**Fig. 2:**
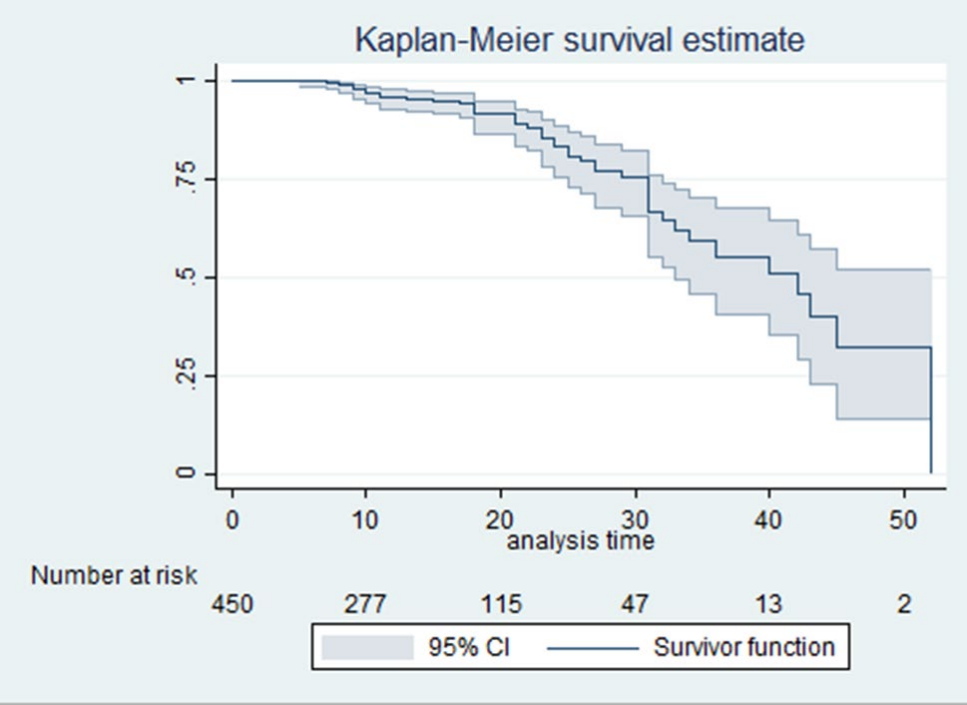
Overall cumulative survival curve of AKI among TBI patients at TGSH, 2022

**Fig. 3:**
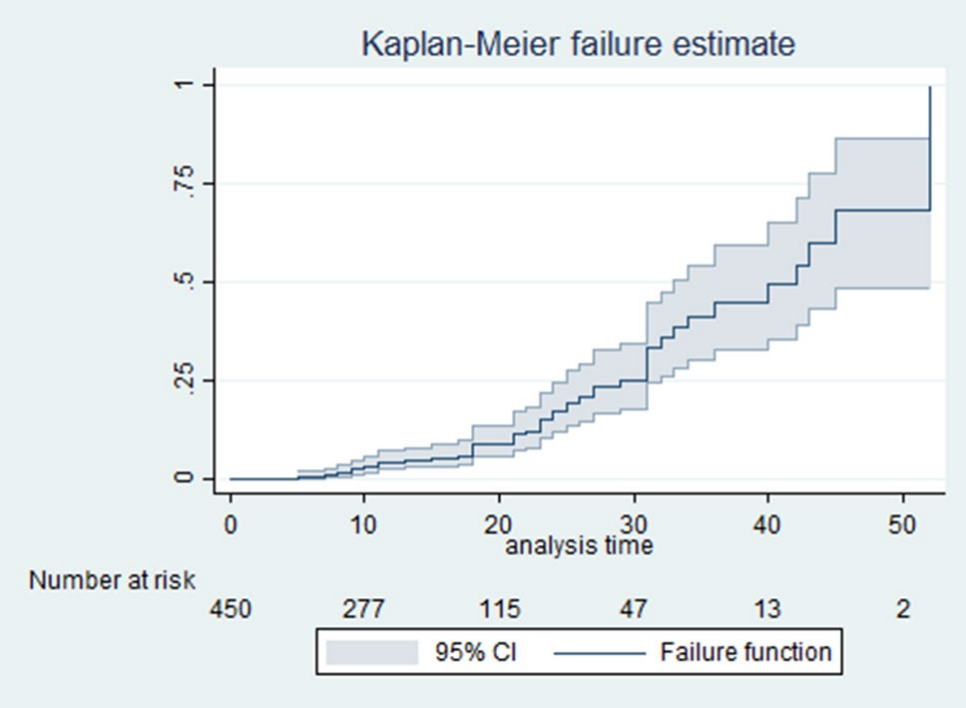
shows overall cumulative hazard curve of AKI among TBI patients at TGSH, 2022

The overall cumulative survival of the TBI patients was shown in the Fig. 2; as illustrated in the graph has a decreasing function indicating the probability of event free days is decreasing. Similarly, the overall hazard rates of TBI patients were shown in the Fig. 3; indicating that the hazards of developing the acute kidney injury is increasing with time.

### Incidence of AKI among different groups of TBI patients

The log-rank test was used to assess survival curves of different groups to determine if there are statistically significant differences between them in terms of developing AKI. The test indicated that the incidence rate of AKI varies across different categories of predictors (Table 3).

**Fig. 4:**
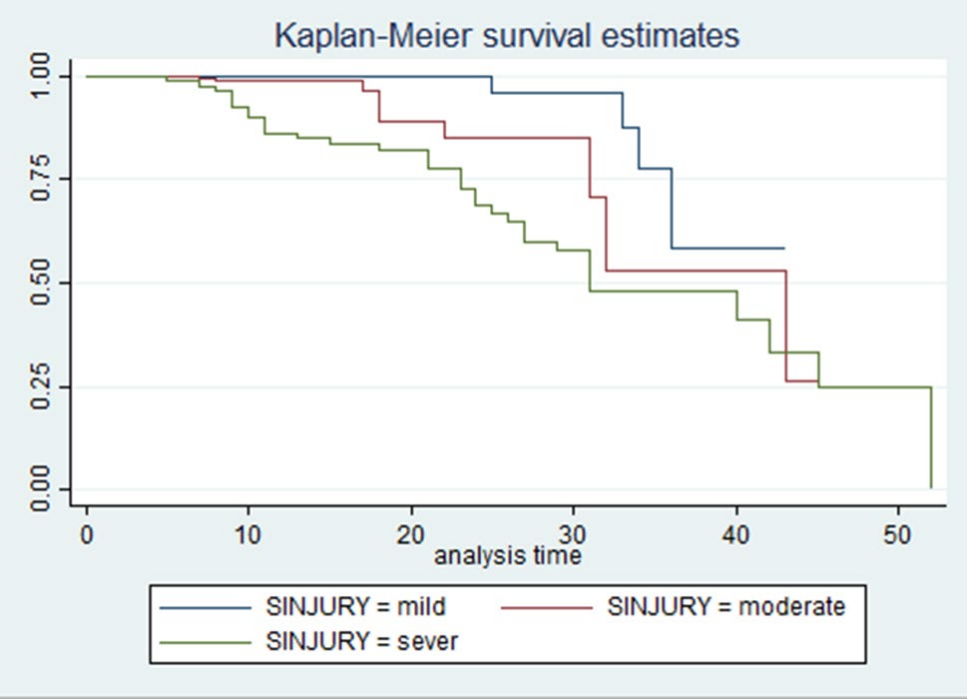
Kaplan Meir survival curves between different levels of injury severity

**Fig. 5:**
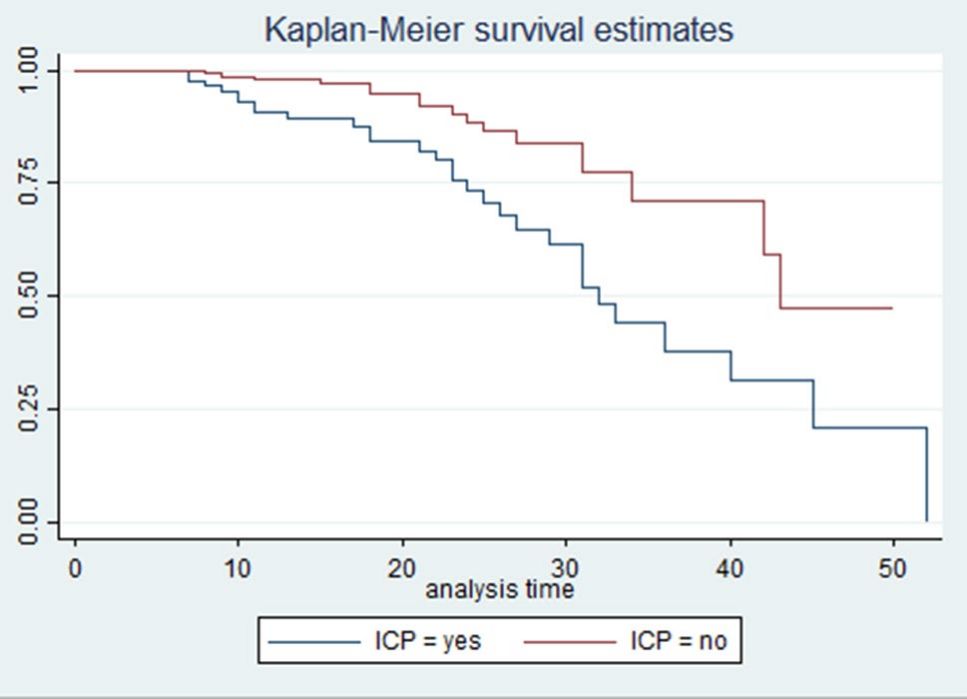
Kaplan-Meir survival curves comparing between patients with ICP and without ICP among post traumatic AKI

Accordingly, the test showed a statistically significant difference for survival times for AKI across levels of injury severity (mild, moderate, and severe), with the mean survival time being lower in patients with severe head injuries compared to those with moderate and mild injuries. This also showed in Kaplan Meir curve analysis (Fig. 4). Additionally, the average survival time to develop AKI was substantially different across the levels of systolic blood pressure; where the mean survival time for hypotensive patients was significantly shorter, with a p-value of < 0.0001. This is also illustrated in Fig. 5.

**Table 3:** Incidence rate, mean survival time (days) and log rank test comparing between different categorical variables of the study

Variable	Category	Incidence rate (95%CI)	Mean (Surv.time) (95%CI)	Log rank
Injury severity	Mild Moderate Sever	0.0017(0.0006–0.0045) 0.0044(0.0024–0.0082) 0.0183(0.0132–0.0256)	46.38(42.85–49.92) 39.91(36.53–43.28) 33.14(29.22–37.05)	0.0001
SBP	Hypotension Normal Hypertension	0.0330(0.0213–0.0511) 0.0045(0.0031–0.0067) 0.0092(0.0034–0.0244)	25.12(20.11–30.13) 42.45(38.96–45.94) 32.29(28.23–36.37)	< 0.0001
Pneumonia (aspiration)	Yes No	0.0217(0.0149–0.0316) 0.0041(0.0027–0.0062)	30.79(26.31–35.26) 41.54(38.36–44.73)	< 0.0001
DM	Yes No	0,0191(0.0127–0.0288) 0.0048(0.0033–0.0071)	31.69(27.65–35.73) 37.36–45.14	0.0056
Pupillary reactivity	Both reactive Both unreactive One reactive	0.0026(0.0015–0.0048) 0.0313(0.0191–0.0501) 0.0117(0.0077–0.0178)	42.19(38.08–46.31) 27.09(20.08–34.10) 35.18(31.77–38.61)	0.0001
Mannitol	Yes No	0.0133(0.0093–0.0189) 0.0044(0.0028–0.0069)	35.80(31.73–39.88) 39.80(35.85–43.76)	0.0352
ICU	Yes No	0.0214(0.0149–0.0306) 0.0037(0.0023–0.0058)	31.96(27.49–36.43) 40.99(37.11–44.88)	0.0001

### Outcomes of patients with AKI

In this study, among the 49 patients who developed acute kidney injury (AKI) following traumatic brain injury (TBI), 26 (53.1%) died, while 23 (46.9%) recovered during their hospital stay. Out of the total patients with AKI, 35 were admitted to the ICU, and 17 (56.7%) of these patients died. Furthermore, 45 AKI cases were reported to have severe or moderate TBI. Within this group 25(55.5%) of the patients with severe or moderate TBI died during the follow-up period (Table 4).

**Table 4:** Outcomes of AKI among selected variables

Variables	Category	AKI Recovered	AKI Died
Injury severity	Mild Moderate Sever	3(75%) 3(30% 17(48.6%)	1(25%) 7(70%) 18(51.4%)
ICU admission	Yes No	13(43.3%) 10(52.6%)	17(56.7%) 9(47.3%)
Pupillary reactivity	Both reactive Both un-reactive One reactive	3(27.3%) 7(43.7%) 13(57.1%)	8(72.7%) 9(56.3%) 9(42.9%)
ICP	Yes No	14(46.7%) 9(47.4%)	16(53.3%) 10(52.6%)
SBP	Hypotension Normal Hypertension	8(40.0%) 13(52.0%) 2(50.0%)	12(60.0%) 12(48.0%) 2(50.0%)
Sex	Male Female	18(51.4%) 5(35.7%)	17(48.6%) 9(64.3%)
Residence	Rural Urban	15(50.0%) 8(42.1%)	15(50.0%) 11(57.9%)

### Analysis of predictors for acute kidney injury (AKI) among traumatic brain injury (TBI) patients using the Cox proportional hazards model

The impact of socio-demographic, interventional, clinical, and patient-related characteristics on the incidence of acute kidney injury (AKI) among traumatic brain injury (TBI) patients was analyzed using the Cox proportional hazards regression model. The predictor variables included in the model were age, pneumonia, injury severity, diabetes mellitus, length of hospital stay, Mannitol use, ICU admission, cerebral hemorrhagic contusion, pupillary reactivity, and systolic blood pressure. The proportional hazards assumptions for these predictor variables were statistically assessed using the Schoenfeld residuals test, indicating that all variables met the PH assumptions (p-value > 0.05). The overall global test yielded a p-value of 0.5048 suggesting a good fit for the model.

**Fig. 6:**
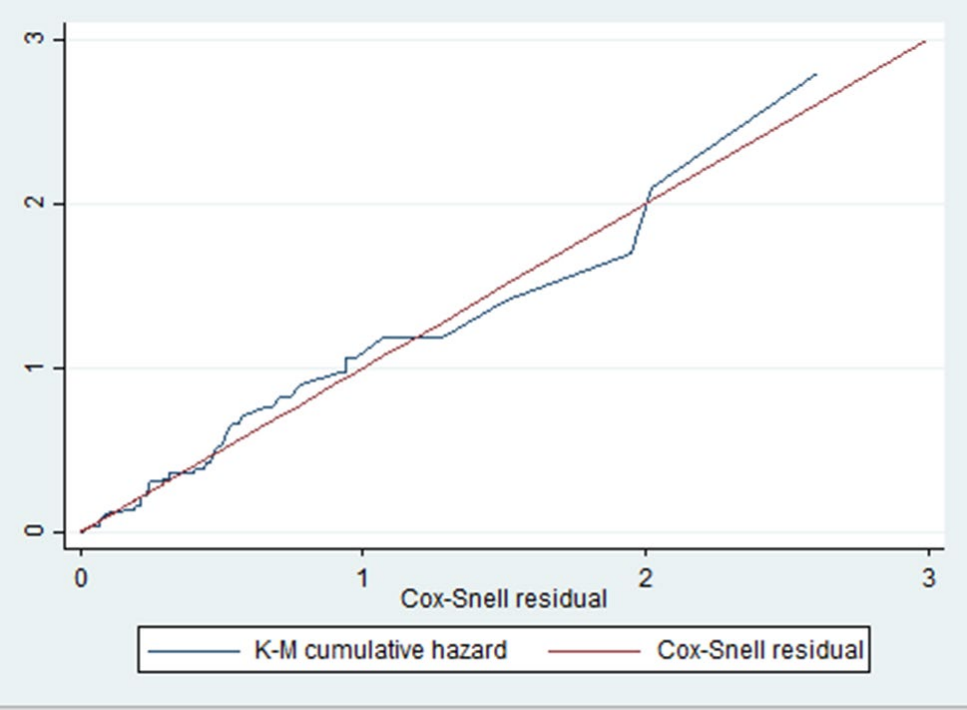
Cox Snell residual test of model fitness of Cox proportional hazard regression model

Additionally, a graphical assessment of the overall model fitness for the data in the Cox proportional hazards regression was assessed using residuals that follow a standard censored exponential distribution with the hazard ratio. When comparing the jagged line to the reference line (Cox-Snell residuals), it was observed that, despite some divergence at the end, the hazard function closely follows the Cox-Snell residual line (Fig. 6).

### Predictors of acute kidney injury among traumatic brain injury patients

Multivariable Cox Regression Analysis in the bivariate Cox regression analysis, several variables emerged as candidates for the multivariable model, including age, injury severity, pupillary reactivity, Mannitol administration, ICU admission, diabetes mellitus, cerebral hemorrhagic contusion, hospital length of stay, and systolic blood pressure. However, the multivariable analysis identified only a few significant predictors of acute kidney injury (AKI). These significant predictors included age (Adjusted Hazard Ratio (AHR): 1.05, 95% Confidence Interval (CI): 1.02–1.07), severe head injury (AHR: 3.85, 95% CI: 1.23–12.03), bilateral unreactive pupils (AHR: 4.65, 95% CI: 1.72–12.53), and hypotension (AHR: 3.15, 95% CI: 1.58–6.29) (Table 5).

**Table 5:** Results of bi-variable and multivariable Cox proportional hazard regression model

Variables	Categories	Cox-regression analysis	
		CHR(95%CI for CHR)	AHR(95%CI for AHR)
Age		1.06(1.03–1.08)	1.05(1.02–1.08)
Injury	Mild	1	1
Severity	Moderate	2.86(0.99–10.28)	1.29(0.87–1.92)
	Sever	6.56(2.31–18.63)	1.46(1.02–2.09)
Pupillary	Both reactive	1	1
Reactivity	Both un-reactive	6.67(3.05–15.04)	4.82(1.82–12.72)
	One reactive	2.92(1.40–6.08)	1.64(0.67–4.02)
Mannitol	Yes	1.85(1.03–3.35)	1.11(0.55–2.26)
	No	1	1
ICU	Yes	3.15(1.75–5.91)	0.96(0.39–2.33)
	No	1	1
DM	Yes	2.45(1.34–4.45)	0.87(0.41–1.83)
	No	1	1
Systolic	Hypotension	5.55(3.04–10.13)	3.45(1.71–6.96)
Blood pressure	Normal	1	1
	Hypertension	1.86(0.64–5.40)	1.31(0.41–4.23)
Pneumonia	Yes	3.62(2.04–6.45)	1.78(0.77–4.11)
(Aspiration)	No	1	1
Cerebral	Yes	2.35(1.32–4.18)	0.87(0.41–1.84)
Contusion	No	1	1
Length of Hospital stay		1.03(1.01–1.05)	0.97(0.94–1.01)

## Discussions

The primary reason for studying individuals with traumatic brain injury (TBI) is that their risk of acute kidney injury (AKI) is higher than compared to those with other types of injuries. This is a plausible hypothesis for this investigation; evidences revealed that approximately 10–39.3% of TBI patients develop AKI, with significant implications for their overall health outcomes [22, 23]. The activation of the sympathetic nervous system (SNS) in traumatic brain injury (TBI) cases contributes to the development of acute kidney injury (AKI). This activation leads to increased levels of catecholamines and dysregulation of immune responses, exacerbating renal damage. The SNS response to TBI is associated with elevated inflammatory responses, which contribute to kidney injury through mechanisms such as oxidative stress and immune dysregulation [24, 25].

Acute kidney injury (AKI) is a critical global health challenge that significantly contributes to morbidity and mortality, particularly in resource-limited settings [26, 27]. The Sustainable Development Goals (SDGs), particularly Goal 3, aim to ensure healthy lives and promote well-being for all at all ages. Within this framework, addressing AKI is crucial, as it aligns with the broader targets of reducing premature mortality from non-communicable diseases and achieving universal health coverage [28]. Improving the early detection and management of AKI, we can contribute to the SDG target of reducing deaths and ensuring that health services are accessible, equitable, and effective for all, especially for vulnerable populations such as those affected by traumatic brain injury. Accordingly, this study aims to investigate the incidence of AKI among TBI patients during their hospital stay, to estimate the time to the occurrence of AKI, and identify predictors of AKI in patients with traumatic brain injury.

The current study revealed that the cumulative incidence rate of acute kidney injury (AKI) among traumatic brain injury (TBI) patients was 10.9% (95% CI: 8.32–14.13), with the highest incidence occurring within the first month after admission. The median survival time was 42 days (IQR: 31–52). This finding is lower compared to a study conducted in Addis Ababa, Ethiopia, which reported an incidence rate of 27.6% [29]. The discrepancy may be attributed to differences in sample size and study design. Similarly, the incidence rate observed in our study is lower than that reported in a study from South Africa [30], potentially due to differences in the study settings. However, our finding is higher than that of a study conducted in Uganda, which reported an incidence rate of 4.8% [31]. This variation could be due to differences in socio-demographic characteristics, study design (cross-sectional), and sample size. Our results align with studies conducted in Spain (10.6%), India (11.8%), and Australia (9.2%) [7, 15, 32]. Nonetheless, the cumulative incidence rate observed in our study is lower than that reported in a retrospective cohort study from China, which found an incidence rate of 19.8% [33]. This relative discrepancy could be due to differences in the socio-demographic characteristics of the study populations and the diagnostic modalities used.

The current study also assessed predictors of AKI; related to socio-demographic, clinical, and patient related characteristics. As a result, the variable age, injury and systolic blood pressure were significant predictors for the occurrence of AKI. For a unit increase in age, will increase the hazard of having AKI among TBI by 5% (AHR; 1.05(1.02–1.07)). This result is in line with studies conducted in china and Australia [7, 15]. The relation may be related to physiologic change in renal function and presence of multiple co-morbidities with age increases. As age increases, kidney will undergo age related structural and functional changes which leads to a reduction in functioning nephron mass and significant decrease in kidney function [34].

Furthermore, the current study also found that the hazard ratio of reporting AKI was more than threefold times higher in patients with hypotension than patients with normal blood pressure (AHR; 3.45(95%CI: 1.71–6.96)). When the body has hypotension, there will be reduced blood flow to the kidney (decreased renal perfusion) which leads to reduction in kidney function. When compared with mild head injury patients, the hazard of having AKI is 1.46 times higher in severe head injury patients (AHR; 1.46(95%CI; 1.02–2.09)).This is due to brain injury affects kidney function with Neuro- endocrine path ways. The increased visceral sympathetic nervous system activation results in reduced renal glomerular perfusion with increased renal sodium reabsorption and subsequent development of AKI among TBI patients [35].

### Strengths and limitations

This study contributes valuable insights into the survival rates and predictors of acute kidney injury (AKI) among traumatic brain injury (TBI) patients in a low-income setting, an area with limited existing research. This focus on a high-risk, underserved population enhances the study’s relevance and potential impact on clinical practice. Furthermore, this study used actual health record data collected at the hospital level, providing a practical and real-world perspective. Such a study design enhances the generalizability of the findings and ensures that the results are directly applicable to clinical practice, offering valuable insights for healthcare providers. However, limitations should be considered when interpreting the results. The retrospective nature of the study posed challenges in obtaining complete patient information, including rhabdomyolysis or creatinine kinase (CK), index of severity of illness (SOFA and APACHE) serum creatinine and urine output. Additionally, vasopressors among patients admitted to the ICU, and certain patient-related factors, such as alcohol consumption and referral systems, were inadequately documented in patient charts, potentially affecting the comprehensiveness of the data.

## Conclusions

This study highlights the magnitudes of acute kidney injury (AKI) among patients with traumatic brain injury (TBI), with a cumulative incidence rate of 10.9% observed over follow-up period. The findings underscore the critical importance of early detection and management of AKI, particularly within the first month after TBI, to improve patient outcomes. Key predictors of AKI, such as age, injury severity, and systolic blood pressure, were identified, emphasizing the need for targeted interventions in these high-risk groups. Despite its limitations, including the retrospective design, this study provides valuable insights into AKI incidence and its predictors in TBI patients. Further prospective research is recommended to explore the underlying mechanisms, include important predictors that often missed in the secondary data, and improve clinical outcomes through targeted interventions.

## Data Availability

Data are available from corresponding author upon request.

## Abbreviations

AKI: Acute kidney injury
TBI: Traumatic brain injury
ICU: Intensive care unit
GCS: Glasgow coma scale
Scr: Serum creatinine
BUN: Blood urea nitrogen
CT: Computerized tomography
MRI: Magnetic resonance imaging
AKIN: Acute kidney injury network
RIFLE: Risk injury failure loss end stage
KDIGO: Kidney disease improving global outcome
GFR: Glomerular filtration rate
NGT: Nasogastric tube
AaBET: Addis Ababa burn emergency and trauma
TGSH: Tibebe Ghion specialized hospital
CKD: Chronic kidney disease
